# Psychometric Properties of the Malay Language Version of Knee Injury and Osteoarthritis Outcome Score (KOOS) Questionnaire among Knee Osteoarthritis Patients: A Confirmatory Factor Analysis

**DOI:** 10.5704/MOJ.1707.003

**Published:** 2017-07

**Authors:** MM Zulkifli, AA Kadir, A Elias, KC Bea, AN Sadagatullah

**Affiliations:** Department of Family Medicine, Universiti Sains Malaysia, Kubang Kerian, Malaysia; ^*^Klinik Kesihatan Cheneh, Kemaman, Malaysia; ^**^Department of Orthopaedics, KPJ Johor Specialist Hospital, Johor Bahru, Malaysia; ^***^Department of Orthopaedics, Universiti Sains Malaysia, Kubang Kerian, Malaysia

**Keywords:** osteoarthritis, construct validity, knee-related quality of life, KOOS

## Abstract

**Introduction:** This study aimed to cross-culturally adapt a Malay version of Knee Injury and Osteoarthritis Outcome Score (KOOS) and to evaluate its psychometric properties in patients with knee osteoarthritis (OA).

**Materials and Methods:** The English version KOOS was translated into a Malay version using forward and backward translation process, followed by face validity and content validity. Two hundred and twenty-six knee OA patients attending the Outpatient and Orthopaedic Clinics, Universiti Sains Malaysia Hospital, completed the Malay version KOOS. Construct validity using confirmatory factor analysis and internal reliability assessment were performed.

**Results:** The results showed that the original five-factor model with 42 items failed to achieve acceptable values of the goodness of fit indices, indicating poor model fit. A new five-factor model of 26 items demonstrated acceptable level of goodness of fit (comparative fit index= 0.929, incremental fit index= 0.930, Tucker Lewis fit index= 0.920, root mean square error of approximation= 0.073 and Chisquared/degree of freedom= 2.183) indices to signify a model fit. The Cronbach’s alpha value for the new model ranged from 0.776 to 0.946. The composite reliability values of each construct ranged between 0.819 and 0.921, indicating satisfactory to high level of convergent validity.

**Conclusion:** The five-factor model with 26 items in the Malay version of KOOS questionnaire demonstrated a good degree of goodness of fit and was found to be valid, reliable and simple as an assessment tool for symptoms, pain, activity of daily living, sports and recreational activity and quality of life for Malaysian adults suffering from knee osteoarthritis.

## Introduction

Knee osteoarthritis (OA) is a degenerative joint disease and it is the leading cause of chronic disability at older ages. This condition impacts health in various ways including functional, mental and economic, and the quality of life. In the past, there have been considerable growth in knee-related rating scales designed to measure outcomes from the perspectives of patients. Some of these instruments have been evaluated for reliability, validity and responsiveness^1^.

One of the most widely used subjective knee measurement tools is the Knee Injury and Osteoarthritis Outcome Score (KOOS)^1,2^. The KOOS is to be used in primary OA or post traumatic OA^3^. This tool is based on Western Ontario and McMaster Universities Osteoarthritis Index (WOMAC) and the WOMAC score can be calculated using this tool^4,5^. This instrument covers both the short-term and long-term consequences. It has been translated and culturally adapted into different languages including Singapore English and Chinese, Korean, Persian and Portugese^6–8^.

However, there is no Malay version available at present. We decided to conduct a process of cross-cultural adaptation and validation in order to use this instrument for the Malay speaking patients in Malaysia. The aim of the present study was to translate and culturally adapt KOOS into Malay to suit Malaysian patients with knee osteoarthritis and to test its psychometric characteristics (construct validity and internal reliability) using confirmatory factor analysis (CFA).

CFA is greater to exploratory factor analysis and simple reliability analysis (test-retest and internal consistency reliabilities) in many aspects^9^. CFA is a type of structural equation modeling that is concerned with measurement models^9^. It is useful to use CFA to verify the relationships between items and respective factors as it provides ways to evaluate the fit of the proposed theoretical model to the collected data^9^.

## Materials and Methods

A cross sectional study was conducted among patients who were diagnosed with knee OA between September 2013 and March 2014 in the Outpatient and Orthopaedic Clinic, Universiti Sains Malaysia Hospital, a tertiary teaching hospital in Malaysia. Patients with knee osteoarthritis diagnosed according to the clinical diagnostic criteria of the American College of Rheumatology 1986^10^ and who were able to read in the Malay language were included.

Sample size for CFA depends on the model complexity and basic measurement model characteristics. Hair *et al* have suggested a minimum sample of 100 for a model with five or less latent constructs and more than three items in each latent construct^11^. Convenient sampling was applied and written informed consent was taken. Patients were asked to fill out the Malay version of KOOS and pro forma on sociodemographic data. The study was approved by the Human Research Ethic Committee of Universiti Sains Malaysia.

The KOOS was first developed in 1995 by Ewa M Roos and colleagues at the Departments of Orthopaedics at Lund University, Sweden, and at the University of Vermont, USA^12^. Thus, the American-English and Swedish versions were developed simultaneously^12^. It has been used in men and women from the ages of 14 to 79 years old^12^. The KOOS is a valid, reliable and responsive self-administered instrument. It holds 42 items in 5 separately scored subscales: Pain, other Symptoms, Function in Daily Living (ADL), Function in Sport and Recreation (Sport & Rec), and Knee-related Quality of Life (QOL)^3,12^.

The forward and backward translation was carried out by a group of panels made up of physicians, linguists and bilingual translators proficient in English and Malay independently. The original English version KOOS was independently translated into Malay language by five translators (physicians, linguist and bilingual translator). A team of researchers then discussed and compared the translated version until a consensus was reached on a single adapted Malay version. Then, the Malay version questionnaire was back-translated to English version by another five translators (physicians, linguistics and bilingual translator) who had not seen the original English version. A meeting was held again to compare the Malay version with the original English version. Modifications were made and content validity was checked. Face validity was later assessed based on respondent testing done among 20 knee osteoarthritis patients. They were required to review and comment on the whole questionnaire in terms of presentation, arrangement, clarity and relatedness. Modifications were made based on the comments yielding a final version of the Malay questionnaire.

Items related to each factor (subscales) were combined to produce mean and standard deviations. Distributions of the scores were checked for possible ceiling or floor effect. This was to ensure that the patients were using the full range of possible scores. Assessment of normality and outliers was performed based on the critical ratio (i.e. for skewness and kurtosis to their standard error), Mahalanobis distance and histogram plots^13^. CFA to test for construct validity and reliability analysis were performed to assess the psychometric properties using SPSS version 22.0 and Analysis of Moment Structure (AMOS) software version 21.0.

Construct validity examines the degree to which a scale measures what it intends to measure^14^. CFA was performed to test that the five factors (domains) identified in the original study would be found with this sample of patients. The construct validity was checked with several goodness-of-fit indicators: Comparative Fit Index (CFI), Tucker Lewis Index (TLI), Incremental Fit Index (IFI), Chi-squared/degree of freedom and Root Mean Squared Error of Approximation (RMSEA)^9,15^. A value of more than 0.9 was taken for CFI, IFI and TLI^15,16^. Chi-squared/degree of freedom of less than 3 and RMSEA value of less than 0.08 was taken as an indicator of acceptable level^9,13^.

The standardized factor loading (standardized regression weight), modification indices (MI), squared multiple correlation (R2) and factor loadings were used as indicators to select which items were fit to be removed in the model^9^during CFA. MI suggested correlations between variables and the factor loadings was used to assess for unidimensionality of the questionnaire^9,13^. Unidimensionality indicates that various items measured the same attitude or ability^13^. For an established questionnaire, the factor loading for each item should be 0.6 or higher^13^.

In addition, Average Variance Extracted (AVE) was used to assess convergent validity and also reliability^13^. AVE is the average percentage of variation explained by the variables in the construct or domain^13^. The acceptable value for it was taken as more than 0.5^13^. Reliability analysis was measured using Cronbach’s alpha coefficient, CR and AVE^11,13^. Cronbach’s alpha coefficient value of more than 0.7 and CR more than or equal to 0.6 represent a measure of satisfactory internal consistency^11,13^.

## Results

The sociodemographic characteristics of the respondents were as shown in Table I. A total of 226 knee osteoarthritis patients had responded. The mean age of the subjects was 50.8 years old. Majority of the respondents were female (79.6%), ethnic Malay (95.6%) and the mean duration of knee osteoarthritis was 3.2 years.

**Table I:** Socio-demographic and clinical characteristic of knee OA patients

**Variables**	**Mean**	**SD**	**N (%)**
Age (year)	50.8	6.3	
Gender			
Male			46 (20.3)
Female			180 (79.6)
Race			
Malay			216 (95.6)
Others			10 (4.4)
Duration of knee OA	3.2	2.5	

Normality assessment showed that the data were normally distributed and the value for skewness for all items was satisfied (0.2–1.1). Table II represents the means, standard deviations (SD), range and proportion of patients scoring at the floor (zero) and the ceiling (100) levels on the 0-100 scale for the KOOS questionnaire. The proportion of patients who had floor effect were negligible for KOOS Symptoms, Sport/Recreational and QOL. There were no ceiling effects for all the domains. There were no missing data of KOOS item.

**Table II:** The mean scores, standard deviations, score ranges and the number (%) of subjects reporting worst possible score (floor effect) and best possible score (ceiling effect) for the Malay version KOOS (n = 226)

	**Mean**	**SD**	**Range**	**Floor effect**	**Ceiling effect**
				**n (%)**	**n (%)**
KOOS Symptoms	12	5.8	0-30	2 (0.9)	0 (0)
KOOS Pain	12	6.4	2-36	0 (0)	0 (0)
KOOS ADL	18	11.5	2-55	0 (0)	0 (0)
KOOS Sport/ Recreation	15	7.7	0-36	2 (0.9)	0 (0)
KOOS QOL	17	6.7	0-33	2 (0.9)	0 (0)

The Malay version KOOS was well accepted in the face validity except for minimal difficulty to understand items s7 and s8 in the questionnaire. Two patients had difficulty understanding item s7 and s8 (“*kekejangan*” which means “cramps or spasm”, to be replaced by “*kekakuan*” which means “stiffness”). Patients commented that there were two questions which they believed were not suitable to them: item a9 and a11 (no knee pain while wearing or removing the socks). The expert panel decided to choose “*kekakuan*” for “stiffness” in item s7 and s8. The original 5-factor model of the Malay version KOOS is shown in Table III.

**Table III:** The original 5-factor model of the Malay version KOOS

**No.**	**Items**	**Coding**
1	**Adakah terdapat bengkak pada sendi lutut anda?**	**s1**
	Do you have swelling in your knee?	
2	**Adakah anda berasa kisaran/ geseran, dengar bunyi klik/ retakan atau bunyi lain apabila**	**s2**
	**sendi anda bergerak?**	
	Do you feel grinding, hear clicking or any other type of noise when your knee moves?	
3	**Adakah sendi lutut anda kejang/ terkunci apabila bergerak?**	**s3**
	Does your knee catch or hang up when moving?	
4	**Bolehkah anda meluruskan sendi lutut anda sepenuhnya?**	**s4**
	Can you straighten your knee fully?	
5	**Bolehkah anda membengkokkan sendi lutut anda sepenuhnya?**	**s5**
	Can you bend your knee fully?	
6	**Berapa terukkah kekakuan sendi lutut sebaik sahaja bangun daripada tidur?**	**s6**
	How severe is your knee joint stiffness after first wakening in the morning?	
7	**Berapa terukkah kekakuan sendi lutut anda selepas duduk, terbaring, atau berehat**	**s7**
	**pada lewat petang?**	
	How severe is your knee stiffness after sitting, lying or resting later in the day?	
8	**Berapa kerapkah anda mengalami kesakitan sendi lutut?**	**p1**
	How often do you experience knee pain?	
9	**Memusing/ memutar sendi lutut**	**p2**
	Twisting/pivoting on your knee	
10	**Luruskan sendi lutut sepenuhnya**	**p3**
	Straightening knee fully	
11	**Bengkokkan sendi lutut sepenuhnya**	**p4**
	Bending knee fully	
12	**Berjalan atas permukaan datar**	**p5**
	Walking on flat surface	
13	**Naik/ turun tangga**	**p6**
	Going up or down stairs	
14	**Pada waktu malam semasa di atas katil**	**p7**
	At night while in bed	
15	**Duduk atau terbaring**	**p8**
	Sitting or lying	
16	**Berdiri tegak**	**p9**
	Standing upright	
17	**Turun tangga**	**a1**
	Descending stairs	
18	**Naik tangga**	**a2**
	Ascending stairs	
19	**Bangun daripada duduk**	**a3**
	Rising from sitting	
20	**Berdiri**	**a4**
	Standing	
21	**Bongkok ke lantai/ mengutip sesuatu benda**	**a5**
	Bending to floor/pick up an object	
22	**Berjalan atas permukaan datar**	**a6**
	Walking on flat surface	
23	**Memasuki/ keluar dari kereta**	**a7**
	Getting in/out of car	
24	**Pergi membeli-belah**	**a8**
	Going shopping	
25	**Memakai stoking/ sarung kaki**	**a9**
	Putting on socks/stockings	
26	**Bangun dari katil**	**a10**
	Rising from bed	
27	**Menanggalkan stoking/ sarung kaki**	**a11**
	Taking off socks/stockings	
28	**Terbaring atas katil (memusing badan, mengekalkan posisi lutut**	**a12**
	Lying in bed (turning over, maintaining knee position)	
29	**Memasuki/ keluar daripada mandi**	**a13**
	Getting in/out of bath	
30	**Duduk**	**a14**
	Sitting	
31	**Memasuki/ keluar dari tandas**	**a15**
	Getting on/off toilet	
32	**Kerja rumah yang berat (memindahkan kotak berat, memberus lantai, dll)**	**a16**
	Heavy domestic duties (moving heavy boxes, scrubbing floors, etc)	
33	**Kerja rumah yang ringan (memasak, membersihkan habuk,dll)**	**a17**
	Light domestic duties (cooking, dusting, etc)	
34	**Mencangkung**	**sp1**
	Squatting	
35	**Berlari**	**sp2**
	Running	
36	**Melompat**	**sp3**
	Jumping	
37	**Memusing/ memutar sendi lutut anda**	**sp4**
	Twisting/pivoting on your injured knee	
38	**Melutut**	**sp5**
	Kneeling	
39	**Berapa kerapkah anda menyedari masalah sendi lutut anda?**	**q1**
	How often are you aware of your knee problem?	
40	**Adakah anda mengubah cara hidup anda untuk mengelakkan aktiviti yang mungkin**	**q2**
	**mencederakan sendi lutut anda?**	
	Have you modified your life style to avoid potentially damaging activities to your knee?	
41	**Berapa banyakkah kesusahan anda akibat kehilangan keyakinan terhadap lutut anda?**	**q3**
	How much are you troubled with lack of confidence in your knee?	
42	**Secara umum, berapa banyakkah anda mengalami kesusahan akibat keadaan lutut anda?**	**q4**
	In general, how much difficulty do you have with your knee?	

Confirmatory analysis showed that the original five-factor model of the Malay version KOOS (42 items) was not fit (Table IV). Four items (s2, s5, p1, q1) were removed one by one due to low factor loadings, as shown in model I. Further deletion was done (s4, p6, p4, p2, a3) due to low factor loading and high MI. High MI indicates that the respective items are redundant. (model II). Two items were set as free parameter estimates (sp2-sp3) based on high MI in model III. Further item deletion was done due low factor loading and high MI (a10, a16, a1, a2, a5, a9, a11) until the goodness-offit indicators of the final model which consist of 26 items (5-factor model) showed that the model was fit. The goodness of fit indices indicated that the final model had a good construct (CFI = 0.929, TLI = 0.920, IFI = 0.930 and chisquared/degree of freedom = 2.183 and RMSEA = 0.073) (Table IV).

**Table IV:** Fitness level of models

**5 Factor Model**	**RMSEA**	**CFI**	**IFI**	**TLI**	**X^2^/df**	**Actions taken**
Original:	0.101	0.767	0.769	0.752	3.282	
(42 items)						
Model I:	0.102	0.790	0.791	0.774	3.359	Delete:
38 items						s2, s5, p1, q1
Model II:	0.970	0.837	0.838	0.825	3.126	Delete:
33 items						s4, p6, p4 p2, a3
Model III	0.085	0.887	0.888	0.875	2.623	Set as free parameters:
30 items						sp2-sp3
						Delete:
						a10, a16, a1
Final model:	0.073	0.929	0.930	0.920	2.183	Delete:
26 items						a2, a5, a9, a11

CFI : Comparative Fit Index

TLI : Tucker Lewis Index

IFI : Incremental Fit Index

X^2^/df : Chi-squared/Degree of freedom

RMSEA : Root Mean Squared Error of Approximation

The final model consists of five constructs: symptoms (four items), pain (five items), ADL (nine items), sport and recreation (five items) and QOL (three items) (Table V) The standardized factor loadings were from 0.6 to 0.8, indicating that all items contributed highly to the construct measures.

**Table V:** Reliability and confirmatory factor analysis of the Malay version KOOS

**Construct**	**Item**	**Factor loading**	**Cronbach’s alpha (above 0.7)**	**CR (above 0.6)**	**AVE (above 0.5)**
Symptom	S1	0.625	0.776	0.785	0.47
	S3	0.707			
	S6	0.727			
	S7	0.693			
Pain	P3	0.722	0.871	0.88	0.59
	P5	0.855			
	P7	0.807			
	P8	0.751			
	P9	0.673			
ADL	A4	0.820	0.946	0.95	0.67
	A6	0.854			
	A7	0.765			
	A8	0.794			
	A12	0.806			
	A13	0.896			
	A14	0.840			
	A15	0.829			
	A17	0.717			
Sport	SP1	0.828	0.932	0.92	0.71
	SP2	0.801			
	SP3	0.833			
	SP4	0.842			
	SP5	0.908			
QOL	Q2	0.803	0.900	0.90	0.76
	Q3	0.930			
	Q4	0.879			

CR : Construct reliability

AVE: Average variance extracted

The reliability analysis showed that the Cronbach’s alpha coefficient value for each construct was greater than 0.7 (Table V). The CR and AVE of each construct also showed that the final construct had a good measure of reliability (Table V).

## Discussion

This is the first study of cross cultural adapting KOOS into Malay version using confirmatory factor analysis. The present study was successfully translated and validated the Malay version of KOOS questionnaire. The Malay translated version was found to be equivalent to the English version. We did not face any major challenges in translating and adapting the English language into the Malay language. However, the two issues were raised about the suitability of the word in two items and the appropriateness of items related to knee pain. This suggest that the Malay version is applicable for use in Malaysian knee osteoarthritis patients.

The Malay version of KOOS was well accepted and demonstrated acceptable psychometric properties with good construct validity in Malaysian patients with knee osteoarthritis. There were a few items that were removed from the domain. However, the results revealed that the final Malay version of KOOS demonstrated good degree of goodness-of-fit and is a reliable assessment tool for knee osteoarthritis in Malaysia.

This is the first study that used confirmatory analysis in the validation analysis of KOOS. CFA is used to verify the factor structure of a measurement instrument. CFA has become more commonly used for construct validation and to provide evidence for convergent and discriminant validity of the theoretical construct^17^. Most of the items were removed because of low factor loading and significant overlapping (high MI). Removal of these items was shown to improve the fit indices of the model, indicating that perhaps they poorly represented the construct being measured. However, the panel of this study had also reviewed the items before they were removed as they might represent important and meaningful construct as mentioned in a previous validation study. According to Zainuddin *et al*, the reliability in CFA is measured by construct reliability, average variance extracted and Cronbach’s alpha^13^. Therefore, in this study, the three analyses of CR, AVE and Cronbach’s alpha are adequate to measures reliability.

Several limitations were encountered. This study was validated among knee osteoarthritis patients in a North East Malaysian state and the findings may also be valid in other states in the country. However, because a large number of items were removed during the study, the KOOS should be administered with caution until cross-validation studies are conducted in other states. Hair *et al* recommended collection of a new sample and validation upon removal of more than 20 percent of items in a questionnaire^11^. Another limitation was that this study did not correlate KOOS with other instruments. Therefore, we recommended further research to validate the Malay version of KOOS in other Malaysian states and to correlate it with other instruments such as the Malay version of WOMAC questionnaire.

In conclusion, the five-factor model with 26 items Malay version of KOOS questionnaire demonstrated a good degree of goodness-of-fit and was found to be highly valid and reliable as an assessment tool for symptoms, pain, activity of daily living, sports and recreational activity and quality of life for Malaysian adults suffering from knee osteoarthritis. This questionnaire is considered potentially very useful during an outpatient visit as a quick assessment of knee pain and may also be used to monitor changes of activities in patient with knee osteoarthritis.

## Abbreviations

ADL,: function in daily living;
Sport & Rec,: sport and recreation;
QOL,: quality of life;
CFA,: confirmatory factor analysis;
CFI,: comparative fit index;
TLI,: Tucker Lewis index;
IFI,: incremental fit index;
RMSEA,: root mean squared error of approximation;
MI,: modification indices;
R^2^,: squared multiple correlation;
AVE,: average variance extracted;
CR,: construct reliability.
